# Effect of Inpatient Cardiac Rehabilitation Combined with Waon Therapy on Exercise Capacity in Elderly Patients with Heart Failure: A Pilot Study

**DOI:** 10.1298/ptr.E10322

**Published:** 2025-05-09

**Authors:** Kazuya YAMAMOTO, Takumi NODA, Koichi ITO, Hiroyuki MIURA, Makoto MURATA, Chiaki YOKOTA

**Affiliations:** 1 Department of Stroke and Cardiovascular Rehabilitation, National Cerebral and Cardiovascular Center, Japan; 2 Department of Cardiovascular Medicine, National Cerebral and Cardiovascular Center, Japan

**Keywords:** Waon therapy, Cardiac rehabilitation, Acute heart failure, Exercise capacity, Elderly patients

## Abstract

Objectives: Inpatient cardiac rehabilitation (CR) and pharmacotherapy are important for better in-hospital outcomes in elderly heart failure (HF) patients. We aimed to examine whether conventional CR combined with Waon therapy (Waon-CR) improves exercise capacity compared to CR alone in elderly HF patients. Methods: Decompensated and hospitalized HF patients who could not walk independently over 200 m were recruited. Patients admitted from May 2020 to March 2021 and from April 2021 to March 2024 were included in the CR and Waon-CR groups, respectively. Participants underwent a 5-session program during hospitalization. The main outcome measure was a 6-minute walk distance (6MWD) at the completion of the program. We also investigated exercise-related adverse events. Results: A total of 34 patients (mean age 79.5 years, 13 males) were enrolled, including 18 patients in the CR group and 16 patients in the Waon-CR group. The 6MWD after the 5-session program was longer in the Waon-CR group than in the CR group (362.2 ± 103.7 vs. 286.3 ± 100.6 m, p = 0.038). Significant improvement of the 6MWD was demonstrated in the Waon-CR group after adjusting for confounding factors (adjusted B, 147.0 m; 95% confidence interval, 41.3–252.8 m, p = 0.012). There were no adverse events during the hospital stay. Conclusions: Inpatient Waon-CR was feasible and led to improved 6MWD in elderly HF patients at the completion of the 5-session program.

## Introduction

The number of elderly hospitalized heart failure (HF) patients with frailty and reduced physical function is steadily increasing, and the medical burden for these patients has become a critical societal concern.^1,2)^ However, the hospital stay for HF patients has decreased from 26 to 16 days within a span of 9 years.^3)^ These patients often do not get enough cardiac rehabilitation (CR) time to improve their physical function during hospitalization. As a result, approximately 37% of elderly patients with HF are discharged before fully regaining their activity levels for daily living, with decreased motor function such as hospitalization-associated disability (HAD).^4)^ Thus, a short-term and effective in-hospital CR program is urgently needed.

In previous reports, CR programs for HF, which improved exercise capacity and re-hospitalization rates, were mainly designed for 3–5-month outpatient CR programs and stable HF patients.^5–7)^ In inpatient CR programs for HF, only middle-aged and older patients showed improved exercise capacity.^8,9)^ Effective inpatient and elderly CR programs for HF are rarely investigated. Moreover, applying exercise-based CR to elderly patients is often limited by their comorbid sarcopenia, cachexia, and frailty.^10)^

Waon therapy is a unique thermal therapy that is implemented in a dry sauna kept at 60°C using far infrared rays.^11)^ In the multicenter prospective randomized WAON-CHF study,^12)^ Waon therapy that was administered as a 10-time intervention during hospitalization led to significant improvements in the 6-minute walk distance (6MWD), with demonstrated safety in patients with advanced HF. Exercise-based CR alone also reportedly improved the 6MWD in HF patients, and guideline-based CR is strongly recommended as a standard intervention for elderly HF treatment.^13)^

We hypothesized that exercise-based CR combined with Waon therapy (Waon-CR) could augment the effects of exercise-based CR alone in elderly HF patients during hospitalization. The present study aims to evaluate whether exercise-based Waon-CR can improve exercise capacity compared to exercise-based CR alone in elderly hospitalized HF patients.

## Methods

This investigation is a single-center retrospective observational study that was approved by the Ethics Committee of the National Cerebral and Cardiovascular Center (approval no. M30-090-5) and registered with the UMIN Clinical Trials Registry (ID: UMIN000050161). All subjects were offered an opt-out opportunity, with the opt-out documents posted in the CR room and on the hospital website.

### HF participants

Hospitalized patients with HF who were admitted to our hospital for decompensation due to acute or acute-on-chronic HF and participating in CR inpatients were included in the present study. The rehabilitation was initiated in the ward as soon as possible. Within a few days after admission, patients who performed their daily activities with complete independence (e.g., independence in transfer activities and walking in their room) but could not continuously walk for more than 200 m were enrolled as study participants.

Our CR programs are classified based on patients' walking ability at the start of CR. Specifically, patients who can walk continuously for more than 200 m are assigned to a standard CR program, which primarily includes guideline-based aerobic exercises. In contrast, for patients who cannot walk continuously for 200 m^13)^, we provide an individualized CR program tailored to their functional status. This study focused on patients who could not walk continuously for more than 200 m.

The patients who underwent exercise-based CR from May 2020 to March 2021 were included in the CR group. Since our hospital began administering Waon therapy for HF patients in 2021, the patients who underwent exercise-based Waon-CR from April 2021 to March 2024 were included in the Waon-CR group.

The exclusion criteria for the present study were as follows: (1) patients for whom exercise therapy was contraindicated according to the current guidelines^14)^; (2) patients receiving hemodialysis; (3) patients receiving intravenous inotropic support after admission; (4) patients who were bedridden before admission; (5) patients with severe cognitive impairment; (6) patients scheduled for discharge within a week; (7) patients deemed ineligible by the attending physician; and (8) patients who were denied admission to the CR program.

### Exercise-based CR program

The exercise-based CR for 1 session consisted of resistance and aerobic training during hospitalization in the CR room. The patients underwent 5-minute resistance training with 3 sets, each consisting of 10 repetitions for calf raises and squats with a 30-second rest between each set, using the horizontal bars. Then, they underwent aerobic exercise sessions with the bicycle ergometer (Teras Ergo; SDG, Tokyo, Japan) at a 5–20 Watt load. The intensity in the initial 10-minute session was adjusted for a low-intensity load (Borg scale 10–11),^15)^ followed by a maximum 15-minute session gradually going up to 12–13 on the Borg scale. After completing the session on the bicycle ergometer, patients walked on the track for 50–100 m at a comfortable speed with intermittent breaks for 15–20 minutes, under the supervision of a physical therapist.

### Waon therapy

**Fig. 1.**
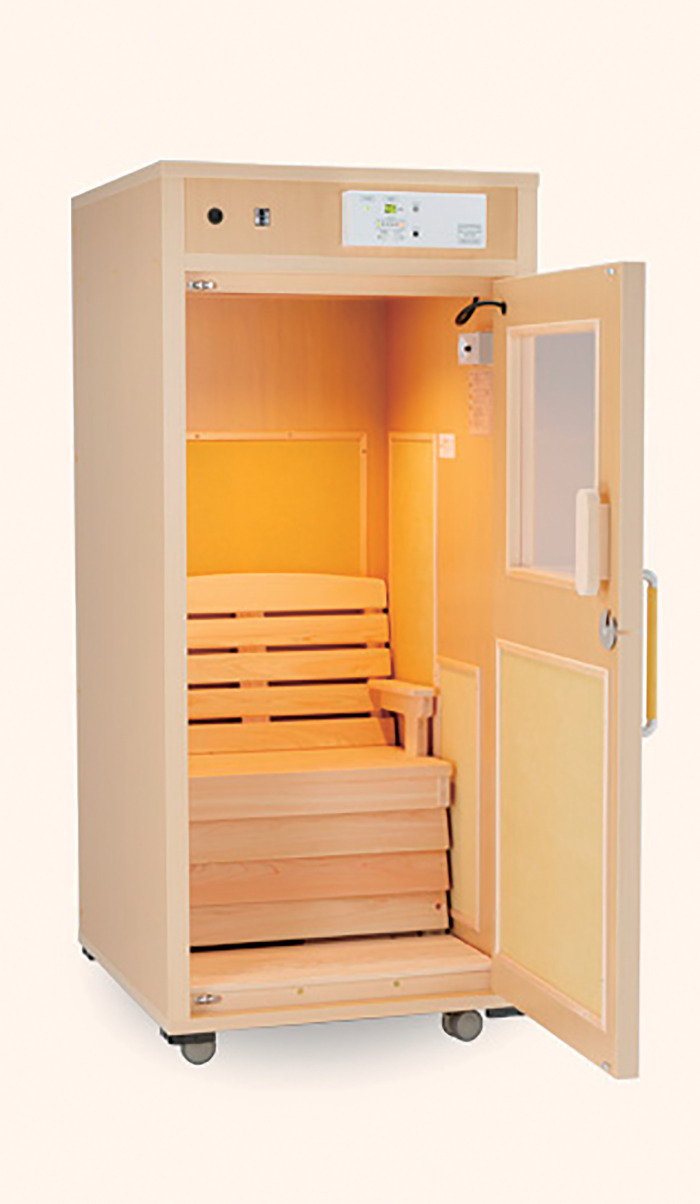
The equipment for Waon therapy. The image is reproduced with permission from Fukuda Denshi (Tokyo, Japan).

Waon therapy uses a far infrared ray dry sauna (CTW-5000; Fukuda Denshi, Tokyo, Japan) that is uniformly maintained at 60°C (Fig. 1). The treatment in our study was conducted as previously reported.^12)^ In brief, patients underwent a 15-minute sauna session inside the Waon equipment, followed by 30 minutes of lying on their back under a warm blanket on the bed outside. Vital signs and body weight were examined before and after Waon therapy. Oral hydration with cold water was used to compensate for the weight loss caused by sweating during Waon therapy.

In the Waon-CR group, the exercise-based Waon-CR was conducted during hospitalization, with Waon therapy in the morning and exercise-based CR in the afternoon of the same day.

### Baseline characteristics

At the start of the CR program, we collected baseline characteristics from medical charts, including sex, age, body mass index (BMI), days from admission to the start of CR, left ventricular ejection fraction as evaluated by echocardiography, New York Heart Association (NYHA) classification on admission, and laboratory data, such as B-type natriuretic peptide and creatinine levels. We also reviewed the medication profiles at the start of CR. The nutritional status on admission was assessed using the Controlling Nutritional Status (CONUT) score, which ranges from 0 to 12, with higher scores indicating worse nutritional status.^16)^

### Outcome measures

The 6MWD was assessed according to the guidelines of the American Thoracic Society^17)^ at the end of the 5-session CR program and was the main outcome measure of the present study. Briefly, it was conducted on a 30-m course in an indoor setting under supervision. Patients were instructed to walk as far as possible for 6 minutes at their own pace, with verbal encouragement provided every minute according to the standard protocol. The total distance walked was recorded in meters.

The isometric knee extension muscle strength (IKEMS) and short physical performance battery (SPPB) were also assessed. Changes in IKEMS (%ΔIKEMS) and SPPB (ΔSPPB) from baseline to program completion were evaluated as well.

For IKEMS, patients sat upright on a bench with knee joints fixed at 90° flexion, and strength was measured twice on each side using a dynamometer. The average value (kgf/kg) was recorded.^18)^ The SPPB comprised a balance test (side-by-side, semi-tandem, and tandem), a 4-m walking speed test, and a 5-chair-stands test, with total scores ranging from 0 to 12 (higher scores indicate better function).^19)^

Additionally, we collected data about CR program-related adverse events from the medical charts, including falls, deterioration in NYHA classification, cardiovascular events, and any deaths.

Although we could not evaluate the 6MWD at baseline due to the reduced physical function of the patients, we assessed the abovementioned outcome parameters at baseline and after 5 sessions in the CR room for both the CR and Waon-CR groups.

### Statistical Analysis

All statistical analyses were performed using RStudio statistical software, version 4.2 (R Foundation for Statistical Computing, Vienna, Austria, https://www.R-project.org). For baseline characteristics, continuous variables were presented as means and standard deviations if normally distributed and expressed as medians with interquartile ranges if they were non-normally distributed. Categorical variables were expressed as counts and percentages. The χ^2^-test, Student’s t-test, and Wilcoxon rank-sum test were used when appropriate to determine differences in patient characteristics between the 2 groups. We used multiple linear regression models adjusted for age, sex, and variables that were significantly different between the 2 groups to estimate the effect of the Waon-CR on outcome measures. A significance level of p <0.05 was considered as statistically significant.

## Results

### Baseline characteristics

In total, 18 out of 194 patients in the CR group and 16 out of 540 patients in the Waon-CR group were enrolled in this study, respectively. The mean age was 79.5 years, and 13 patients were male. The median SPPB was 9.5 [7.0, 12.0]. The CR program was initiated at 4.5 days after admission in both groups. The median length of the hospital stay was 18 days, and there were no significant differences between the 2 groups.

Baseline characteristics are shown in Table 1. 68 percent of participants had HF with preserved ejection fraction. There was no difference between the 2 groups with respect to age, gender, BMI, SPPB, and IKEMS at baseline. The etiology of HF differed for the 2 groups: valvular heart disease was more prevalent in the CR group, and dilated/hypertrophic cardiomyopathy was more prevalent in the Waon-CR group. Regarding medications, only the usage of sodium glucose co-transporter-2 inhibitors (SGLT2Is) was higher in the Waon-CR group. Nutritional status, as assessed by the CONUT score, was worse in the Waon-CR group than in the CR group (4.6 ± 2.0 vs. 3.0 ± 1.7, p = 0.020).

**Table 1.** Baseline characteristics

	Overall (n = 34)	CR group (n = 18)	Waon-CR group (n = 16)	p-value
Age, years	79.5 ± 6.2	79.1 ± 6.6	80.1 ± 5.9	0.642
Male, n (%)	13 (38.2)	5 (27.8)	8 (50.0)	0.183
BMI, kg/m^2^	20.9 ± 2.8	20.1 ± 3.0	21.7 ± 2.3	0.116
Admission to initiation of CR, days	4.5 [3.0, 6.8]	6.0 [3.8, 7.8]	4.0 [3.0, 5.3]	0.204
LVEF (%)	59.5 [43.5, 60.0]	60.0 [46.8, 60.0]	54.5 [43.0, 60.3]	0.848
LVEF ≥50%, n (%)	23 (67.7)	13 (72.2)	10 (62.5)	0.545
NYHA classification ≥ III, n (%)	19 (55.9)	10 (55.6)	9 (56.3)	0.968
Etiology of HF, n (%)				
CAD	2 (5.9)	0 (0.0)	2 (12.5)	0.122
VHD	21 (61.8)	14 (77.8)	7 (43.8)	0.042
DCM/HCM	7 (20.6)	1 (5.6)	6 (37.5)	0.022
Other cardiac disease	4 (11.8)	3 (16.7)	1 (6.3)	0.347
BNP, pg/mL	284.0 [176.0, 565.9]	289.9 [156.4, 513.9]	283.9 [203.0, 623.6]	0.605
Creatinine, mg/mL	1.1 [1.0, 1.5]	1.3 [1.1, 1.5]	1.1 [0.8, 1.3]	0.091
CONUT score	3.7 ± 2.0	3.0 ± 1.7	4.6 ± 2.0	0.020
Medications, n (%)				
Beta-blocker	22 (64.5)	12 (66.7)	10(62.5)	0.800
ACE inhibitor/ARB/ARNI	17 (50.0)	10 (55.6)	7 (43.8)	0.492
MRA	15 (44.1)	10 (55.6)	5 (31.3)	0.154
SGLT2I	8 (23.5)	1 (5.6)	7 (43.8)	0.009
SPPB	9.5 [7.0, 12.0]	9.0 [6.0, 12.0]	10.0 [8.0, 11.0]	0.875
IKEMS, kgf/kg	0.38 ± 0.14	0.37 ± 0.16	0.38 ± 0.12	0.858

Measurements are represented as mean ± SD, median [interquartile range], or number (%).

ACE, angiotensin-converting enzyme; ARB, angiotensin II receptor blocker; ARNI, angiotensin receptor-neprilysin inhibitor; BMI, body mass index; BNP, B-type natriuretic peptide; CAD, coronary artery disease; CONUT, controlling nutritional status; CR, cardiac rehabilitation; DCM/HCM, dilated cardiomyopathy/hypertrophic cardiomyopathy; HF, heart failure; IKEMS, isometric knee extension muscle strength; LVEF, left ventricular ejection fraction; MRA, mineralocorticoid receptor antagonist; NYHA, New York Heart Association; SGLT2I, sodium glucose co-transporter-2 inhibitor; SPPB, short physical performance battery; VHD, valvular heart disease; Waon-CR, CR combined with Waon therapy

### 6MWD, IKEMS, and SPPB

**Fig. 2.**
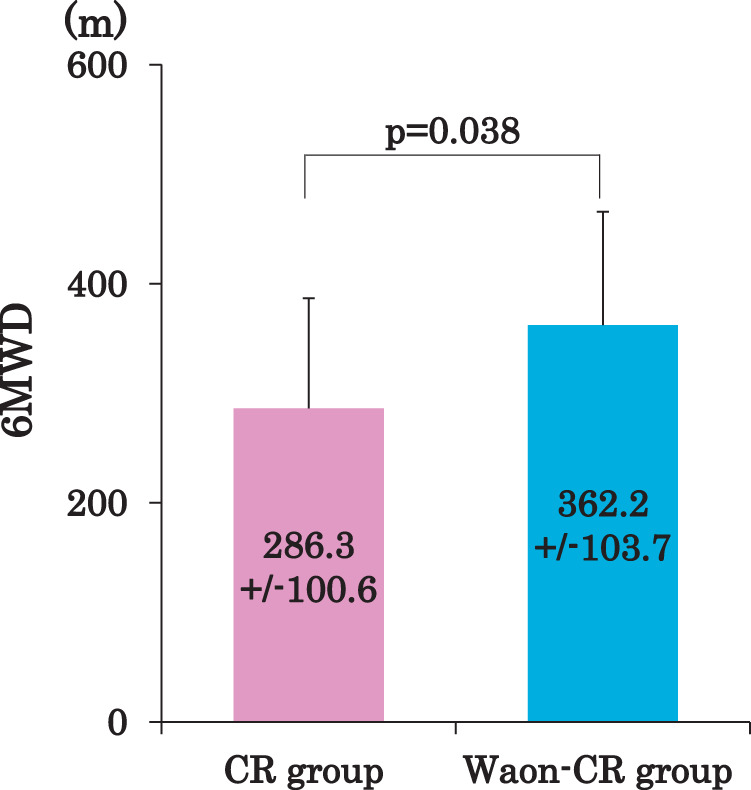
6MWD after the 5-session program of CR. The 6MWD after the 5-session program was significantly longer in the Waon-CR group compared to the CR group (362.2 ± 103.7 vs. 286.3 ± 100.6 m, p = 0.038). Data are presented as mean ± standard deviation. 6MWD, 6-minute walk distance; CR, cardiac rehabilitation.

Regarding the main outcome measure, the 6MWD after CR was longer in the Waon-CR group than in the CR group (362.2 ± 103.7 vs. 286.3 ± 100.6 m, p = 0.038) (Table 2 and Fig. 2). Significant improvements in the 6MWD were seen after 5 CR sessions in the Waon-CR group compared to the CR group, after adjusting for age, sex, etiology of HF, nutritional status (assessed by the CONUT score), and the use of SGLT2Is (adjusted B, 147.0 m; 95% confidence interval, 41.3–252.8 m; p = 0.012).

**Table 2.** Outcome measures

	CR group (n = 18)	Waon-CR group (n = 16)	p-value
6MWD			
After 5 sessions of CR, m	286.3 ± 100.6	362.2 ± 103.7	0.038
SPPB			
After 5 sessions of CR	11.0 [9.0, 12.0]	11.5 [10.0, 12.0]	0.522
ΔSPPB	1.5 [0.0, 2.8]	1.0 [0.0, 2.3]	0.902
IKEMS			
After 5 sessions of CR, kgf/kg	0.37 ± 0.13	0.43 ± 0.11	0.130
% ΔIKEMS, %	−3.1 [−10.3, 10.8]	14.9 [−0.5, 23.4]	0.062

Measurements are represented as mean ± SD or median [interquartile range].

CR, cardiac rehabilitation; 6MWD, 6-minute walk distance; IKEMS, isometric knee extension muscle strength; SPPB, short physical performance battery; Waon-CR, CR combined with Waon therapy

On the other hand, the SPPB and IKEMS after the 5-session CR program were not significantly different between the 2 groups (CR group vs. Waon-CR group; SPPB: 11.0 [9.0, 12.0] vs. 11.5 [10.0, 12.0], p = 0.522; IKEMS: 0.37 ± 0.13 vs. 0.43 ± 0.11, p = 0.130). ΔSPPB and %ΔIKEMS were also not significantly different between the 2 groups.

### Adverse events

There were no CR-related adverse events during the hospital stay in either group.

## Discussion

We demonstrated that, in elderly hospitalized patients with HF and reduced physical function, 5 sessions of exercise-based Waon-CR significantly improved the 6MWD compared to exercise-based CR alone.

The mean age of our HF patients was 79.5 years, and they had reduced physical function (SPPB: 9.5 [7.0, 12.0]). They could not continuously walk for more than 200 m due to their reduced physical function at baseline. In a previous HF study, outpatient CR generally improved physical function, exercise capacity, re-hospitalization rates, and mortality rates.^5–7)^ Kitzman et al. reported that older frail patients exhibited improved SPPB after 3 months of outpatient CR.^20)^ Regarding inpatient CR for HF, Kakutani et al. reported that inpatient CR led to a reduction in hospitalization days in aged patients (80 years old) from 51 to 33 days.^21)^ 2 weeks of CR also led to improved 6MWD in patients (80 years old) with transcatheter aortic valve replacement.^22)^ In the ERIC study, the mean value for hospitalization days was 16, and 12 exercise sessions led to improved 6MWD in decompensated HF patients.^9)^ Oliveira et al. also reported that 10 days of inpatient CR resulted in improved 6MWD.^8)^ However, the mean ages of these patients ranged from 58 to 68 years.

Although there have been several studies on inpatient CR, there are no reports of appropriate CR for elderly HF patients that is tailored to the current average length of hospital stay. Since only 10 sessions of Waon therapy led to improved 6MWD in patients with advanced HF,^12)^ we hypothesized that exercise therapy combined with Waon therapy could augment 6MWD values in elderly HF patients during shorter hospitalization periods.

Our outcome measures were evaluated after the CR program. Therefore, the higher mean value of the 6MWD after the CR program in the Waon-CR group indicates that patients in the Waon-CR group had better improvements in exercise capacity compared to those in the CR group. Waon therapy reduces peripheral vasodilation by enhancing endothelial function via pre- and post-cardiac load reduction^23,24)^ and normalizes cardiac autonomic nervous activity.^25)^ Unfortunately, we did not analyze these functional data. However, it is possible that Waon therapy, in addition to exercise therapy, contributed to the increase in 6MWD through these mechanisms. For example, Waon therapy can dilate the pulmonary artery and improve lung congestion more effectively than exercise alone. These issues therefore warrant further investigation.

It is notable that Waon-CR therapy may be especially effective in elderly hospitalized patients with HF.

Moreover, we found that the baseline nutritional status, as assessed by the CONUT score, was poorer in the Waon-CR group. HF patients with poor nutritional status are known to have reduced physical function.^26)^ On the other hand, SGLT2I usage was higher in the Waon-CR group. SGLT2Is have been recognized as standard medications for HF since 2020–2021 and have been included in various clinical guidelines.^27)^ This likely accounts for their significantly higher usage in the Waon-CR group. A meta-analysis reported that SGLT2Is improved maximal oxygen consumption and the 6MWD in patients with HF.^28)^ Our primary analysis demonstrated that the significant improvement in 6MWD in the Waon-CR group was present even after adjusting for age, sex, etiology of HF, CONUT score, and SGLT2I usage. The observed benefits of Waon therapy combined with CR may be attributed to its unique therapeutic effects rather than differences in nutritional status or medication use.

In the present study, there were no significant differences between the 2 groups with regard to SPPB, IKEMS scores, or their changes from baseline following the completion of the CR program. The median ΔSPPB values in both groups (1.0 point) were above the reported minimal meaningful change (0.5 points),^29)^ indicating that significant clinical improvements in physical function were seen for exercise-based CR with or without Waon therapy. Since the median SPPB at baseline in the Waon-CR group was as high as 10 points in the present study, the benefit of Waon therapy could not be detected due to the ceiling effect of the SPPB. As for the IKEMS, the rapid gains in muscle strength after 5 sessions of CR were relatively higher in the Waon-CR group compared to the CR group, probably due to neural adaptations resulting from increased exercise capacity. Muscle fibers that undergo cellular and molecular changes in HF patients with deconditioning are prone to a slow-to-fast twitch fiber shift,^30,31)^ which could be amenable to neural adaptation. Since physiologic, enzymatic, morphologic, and other muscle-related changes occur after about 30 days of training,^32)^ a longer period might be necessary to observe significant improvements in muscle strength.

Several limitations must be addressed in the present study. First, since the study involved an observational single-center design with a small sample size, generalizing the results is challenging. Our study included 34 participants, as the analysis required 34 patients based on the effect size calculated using the results of a previous study.^12)^ This calculation was performed using following conditions with the G*Power 3.1 program (Heinrich-Heine-Universität, Düsseldorf, Germany): effect size = 0.52, α error probability = 0.05, power (1 − β error probability) = 0.8, and number of predictors = 6 (age, sex, etiology of HF, CONUT score, SGLT2Is, and group). Second, since the study had a retrospective observational design, it was challenging to completely exclude the effects of medication. However, multiple analyses were performed to mitigate this. Third, the optimal structure and duration of Waon therapy combined with CR remain unclear. Although Waon therapy enabled HF patients to participate effectively in exercise-based CR during hospitalization, further investigations are needed to determine ideal inpatient CR programs and the optimal integration of Waon therapy.

Despite its several limitations, the improvement in exercise capacity can be used as a prognostic parameter for mortality and HF re-hospitalization in patients with HF.^7,33,34)^ The development of an optimal program that combines exercise-based CR with Waon therapy is therefore needed to achieve these goals. Our study showed that 5 sessions of CR and Waon therapy were sufficient to increase the 6MWD in elderly hospitalized HF patients with reduced physical function, compared to CR alone. This CR program may have the potential to improve rates for HAD and cardiovascular events during shorter hospitalization periods.

## Conclusions

In conclusion, short-term inpatient Waon-CR improved the 6MWD compared to inpatient CR alone in elderly HF patients. Initiating exercise-based Waon-CR early after admission is feasible and practical, and can lead to improved exercise capacity at discharge for frail HF patients.

## Acknowledgments

The authors sincerely thank Yoichi Goto, MD, PhD, for their significant comments and the physical therapists of the Department of Cerebral and Cardiovascular Rehabilitation in the National Cerebral and Cardiovascular Center for their technical assistance.

## Funding

This study was supported by a Grant-in-Aid for Scientific Research (KAKENHI, 21K11332) from the Ministry of Education, Culture, Sports, Science and Technology of Japan, and by the Intramural Research Fund of the National Cerebral and Cardiovascular Center (24-C-3).

## Conflict of Interest

The authors declare no conflicts of interest.

## References

[1] Mollar A Bonanad C, *et al.*: Frailty and hospitalization burden in patients with chronic heart failure. Am J Cardiol. 2022; 183: 48–54. 36153181 10.1016/j.amjcard.2022.08.013

[2] Lai HY Huang ST, *et al.*: The burden of frailty in heart failure: prevalence, impacts on clinical outcomes and the role of heart failure medications. J Cachexia Sarcopenia Muscle. 2024; 15: 660–670. 38291000 10.1002/jcsm.13412 PMC10995260

[3] Shiraishi Y Kohsaka S, *et al.*: 9-year trend in the management of acute heart failure in Japan: a report from the National Consortium of Acute Heart Failure Registries. J Am Heart Assoc. 2018; 7: e008687. 30371201 10.1161/JAHA.118.008687 PMC6222932

[4] Takahashi T Iwata K, *et al.*: Incidence of hospitalization-associated disability in older patients with heart failure. Circ J. 2024; 88: 672–679. 38220172 10.1253/circj.CJ-23-0722

[5] Molloy CD Long L, *et al.*: Exercise-based cardiac rehabilitation for adults with heart failure - 2023 Cochrane systematic review and meta-analysis. Eur J Heart Fail. 2023; 25: 2263–2273. 37850321 10.1002/ejhf.3046

[6] Belardinelli R Georgiou D, *et al.*: Randomized, controlled trial of long-term moderate exercise training in chronic heart failure: effects on functional capacity, quality of life, and clinical outcome. Circulation. 1999; 99: 1173–1182. 10069785 10.1161/01.cir.99.9.1173

[7] Nakanishi M Miura H, *et al.*: Association of adherence to a 3 month cardiac rehabilitation with long-term clinical outcomes in heart failure patients. ESC Heart Fail. 2022; 9: 1424–1435. 35142087 10.1002/ehf2.13838 PMC8934955

[8] Oliveira MF Santos RC, *et al.*: Safety and efficacy of aerobic exercise training associated to non-invasive ventilation in patients with acute heart failure. Arq Bras Cardiol. 2018; 110: 467–475. 29538506 10.5935/abc.20180039 PMC5967141

[9] Delgado B Novo A, *et al.*: The effects of early rehabilitation on functional exercise tolerance in decompensated heart failure patients: Results of a multicenter randomized controlled trial (ERIC-HF study). Clin Rehabil. 2022; 36: 813–821. 35313751 10.1177/02692155221088684 PMC9082976

[10] Vitale C Jankowska E, *et al.*: Heart Failure Association of the European Society of Cardiology position paper on frailty in patients with heart failure. Eur J Heart Fail. 2019; 21: 1299–1305. 31646718 10.1002/ejhf.1611

[11] Miyata M Tei C. Waon therapy for cardiovascular disease: innovative therapy for the 21st century. Circ J. 2010; 74: 617–621. 20154403 10.1253/circj.cj-09-0939

[12] Tei C Imamura T, *et al.*: Waon therapy for managing chronic heart failure - results from a multicenter prospective randomized WAON-CHF study. Circ J. 2016; 80: 827–834. 27001189 10.1253/circj.CJ-16-0051

[13] Makita S Yasu T, *et al.*: JCS/JACR, 2021 Guideline on rehabilitation in patients with cardiovascular disease. Circ J. 2022; 87: 155–235. 36503954 10.1253/circj.CJ-22-0234

[14] Izawa H Yoshida T, *et al.*: Standard Cardiac Rehabilitation Program for Heart Failure. Circ J. 2019; 83: 2394–2398. 31708554 10.1253/circj.CJ-19-0670

[15] Borg GA. Psychophysical bases of perceived exertion. Med Sci Sports Exerc. 1982; 14: 377–381. 7154893

[16] Ignacio de Ulibarri J Gonzalez-Madrono A, *et al.*: CONUT: a tool for controlling nutritional status. First validation in a hospital population. Nutr Hosp. 2005; 20: 38–45. 15762418

[17] Laboratories ATSCoPSfCPF. ATS statement: guidelines for the six-minute walk test. Am J Respir Crit Care Med. 2002; 166: 111–117. 12091180 10.1164/ajrccm.166.1.at1102

[18] Yokota C Kamada M, *et al.*: Effect of outpatient cardiac rehabilitation on motor function and health-related quality of life in stroke survivors. J Clin Neurosci. 2024; 123: 1–6. 38508016 10.1016/j.jocn.2024.03.013

[19] Guralnik JM Simonsick EM, *et al.*: A short physical performance battery assessing lower extremity function: association with self-reported disability and prediction of mortality and nursing home admission. J Gerontol. 1994; 49: M85–M94. 8126356 10.1093/geronj/49.2.m85

[20] Kitzman DW Whellan DJ, *et al.*: Physical rehabilitation for older patients hospitalized for heart failure. N Engl J Med. 2021; 385: 203–216. 33999544 10.1056/NEJMoa2026141 PMC8353658

[21] Kakutani N Fukushima A, *et al.*: Progressive mobilization program for patients with acute heart failure reduces hospital stay and improves clinical outcome. Circ Rep. 2019; 1: 123–130. 33693126 10.1253/circrep.CR-19-0004 PMC7890289

[22] Kleczynski P Trebacz J, *et al.*: Inpatient cardiac rehabilitation after transcatheter aortic valve replacement is associated with improved clinical performance and quality of life. J Clin Med. 2021; 10: 10. 10.3390/jcm10102125 PMC8156110 34068973

[23] Kihara T Biro S, *et al.*: Repeated sauna treatment improves vascular endothelial and cardiac function in patients with chronic heart failure. J Am Coll Cardiol. 2002; 39: 754–759. 11869837 10.1016/s0735-1097(01)01824-1

[24] Imamura M Biro S, *et al.*: Repeated thermal therapy improves impaired vascular endothelial function in patients with coronary risk factors. J Am Coll Cardiol. 2001; 38: 1083–1088. 11583886 10.1016/s0735-1097(01)01467-x

[25] Kuwahata S Miyata M, *et al.*: Improvement of autonomic nervous activity by Waon therapy in patients with chronic heart failure. J Cardiol. 2011; 57: 100–106. 20884178 10.1016/j.jjcc.2010.08.005

[26] Lelli D Tolone S, *et al.*: Nutritional status is associated with physical function and disability in older adults with chronic heart failure. Eur J Intern Med. 2020; 74: 73–78. 31874803 10.1016/j.ejim.2019.12.007

[27] Tsutsui H Ide T, *et al.*: JCS/JHFS 2021 Guideline focused update on Diagnosis and Treatment of Acute and Chronic Heart Failure. J Card Fail. 2021; 27: 1404–1444. 34600838 10.1016/j.cardfail.2021.04.023

[28] Gao M Bhatia K, *et al.*: SGLT2 inhibitors, functional capacity, and quality of life in patients with heart failurea systematic review and meta-analysis. JAMA Netw Open. 2024; 7: e245135. 38573633 10.1001/jamanetworkopen.2024.5135 PMC11192183

[29] Perera S Mody SH, *et al.*: Meaningful change and responsiveness in common physical performance measures in older adults. J Am Geriatr Soc. 2006; 54: 743–749. 16696738 10.1111/j.1532-5415.2006.00701.x

[30] Mancini DM Coyle E, *et al.*: Contribution of intrinsic skeletal muscle changes to 31P NMR skeletal muscle metabolic abnormalities in patients with chronic heart failure. Circulation. 1989; 80: 1338–1346. 2805270 10.1161/01.cir.80.5.1338

[31] Borina E Pellegrino MA, *et al.*: Myosin and actin content of human skeletal muscle fibers following 35 days bed rest. Scand J Med Sci Sports. 2010; 20: 65–73. 19883388 10.1111/j.1600-0838.2009.01029.x

[32] Kraemer WJ Fleck SJ, *et al.*: Strength and power training: physiological mechanisms of adaptation. Exerc Sport Sci Rev. 1996; 24: 363–397. 8744256

[33] Murata M Yanai S, *et al.*: Improved peak oxygen uptake reduces cardiac events after 3 weeks of inpatient cardiac rehabilitation for chronic heart failure patients. Circ Rep. 2023; 5: 238–244. 37305791 10.1253/circrep.CR-23-0040 PMC10247349

[34] Swank AM Horton J, *et al.*: Modest increase in peak VO2 is related to better clinical outcomes in chronic heart failure patients: results from heart failure and a controlled trial to investigate outcomes of exercise training. Circ Heart Fail. 2012; 5: 579–585. 22773109 10.1161/CIRCHEARTFAILURE.111.965186 PMC3732187

